# Aqueous vascular endothelial growth factor and clinical outcomes correlation after single intravitreal injection of bevacizumab in patients with neovascular age-related macular degeneration

**DOI:** 10.1186/s40942-017-0066-y

**Published:** 2017-05-01

**Authors:** Thiago Cabral, Luiz H. Lima, Júlia Polido, Jimmy Duong, Érika Okuda, Akiyoshi Oshima, Pedro Serracarbassa, Caio V. Regatieri, Rubens Belfort

**Affiliations:** 1 Federal University of Sao Paulo (UNIFESP), Rua Botucatu, 821, Vila Clementino, São Paulo, SP 04023-062 Brazil; 2 Department of Ophthalmology, Columbia University, New York, NY USA; 3 Federal University of Espírito Santo (UFES), Vitoria, Brazil; 4 Department of Biostatistics, Columbia University, New York, NY USA; 5 Public Server Hospital of São Paulo (IAMSPE), São Paulo, Brazil

**Keywords:** Age-related macular degeneration, Aqueous humor, Bevacizumab, Choroidal neovascularization, Vascular endothelial growth factor

## Abstract

**Purpose:**

To evaluate the concentration of vascular endothelial growth factor (VEGF) in aqueous humor after a single intravitreal injection of bevacizumab (IVB) in eyes with neovascular age-related macular degeneration (AMD).

**Methods:**

In this prospective interventional case series study, 24 eyes of 24 patients with types 1 and 2 choroidal neovascularization secondary to neovascular AMD were treated with a single intravitreal injection of bevacizumab. Aqueous humor samples were obtained before the intravitreal injection and at one week, one month, and three months follow-up periods. Best-corrected visual acuity (BCVA) and three spectral-domain optical coherence tomography parameters (central retinal thickness, macular volume and macular area) were also analyzed and correlated with VEGF expression at the baseline and each follow-up period.

**Results:**

All of the ninety-six aqueous humor study taps were well tolerated by the study patients without adverse events. Increased VEGF levels (mean ± SD = 179.7 ± 88.3 pg/mL) were observed in the aqueous humor of all study patients before the intravitreal injection of bevacizumab. At all follow-up periods, compared to baseline, levels of VEGF significantly reduced (P < 0.0001), and BCVA significantly improved (P < 0.005). The lowest VEGF expression was observed at 1 week, and the greatest BCVA improvement occurred 1 month after treatment. At 1 month, central retinal thickness (CRT), macular volume (MV), and macular area (MA) significantly reduced compared to baseline (P < 0.0001, P = 0.0005, P = 0.007, P = 0.009, respectively). At 1 week and 3 months, although without statistical significance (P > 0.005), CRT, MV and MA also reduced in comparison to baseline.

**Conclusions:**

Single intravitreal bevacizumab injection in eyes with neovascular AMD resulted in a substantial decrease of aqueous VEGF levels 1 week after treatment with the greatest improvement of clinical outcomes occurring at 1 month follow-up.

## Background

Neovascular age-related macular degeneration (AMD) is the main cause of severe visual loss among individuals older than 55 years in developed countries [1, 2]. Choroidal neovascularization (CNV) secondary to AMD has been demonstrated to be associated with an imbalance of several angiogenesis growth factors, such as vascular endothelial growth factor (VEGF) and platelet-derived growth factor (PDGF) [3–5]. VEGF is a potent cytokine modulator of angiogenesis, promotes the growth of both retinal and choroidal new vessels and is considered critical for the CNV development [6–10].

A variety of randomized and controlled clinical trials have shown that intravitreal injections of VEGF antibodies, either bevacizumab, ranibizumab or aflibercept, are an effective approach of treatment in patients with neovascular AMD [11–13]. These trials demonstrated that anti-VEGF agents are able to decrease the intraocular VEGF level, causing regression of CNV and reducing retinal and choroidal leakage. Anti-VEGF agents are also related to visual acuity improvement and central retinal thickness (CRT) thinning in neovascular AMD patients [11, 13].

Although there is no consensus regarding both the optimal model to measure VEGF in the eye and the relevance of such measurements, several studies have shown that the decrease of VEGF levels in the eye may reflect a positive response to anti-VEGF therapy [14–17]. The levels of VEGF in retina and choroid are supposed to correspond to VEGF expression in the vitreous humor [18–20]. Nonetheless, vitreous taps are risky and and may cause adverse effects such as vitreous hemorrhage, endophthalmitis, lens opacity and retinal detachment. Conversely, samples of aqueous humor are safer and easier to collect, and similar levels of VEGF has been reported in both the aqueous and vitreous humors [21, 22].

The VEGF concentration in the aqueous humor has been observed to be decreased in eyes with diabetic retinopathy 1 week after intravitreal injection of bevacizumab [23]. Also, several reports have demonstrated improvement on clinical outcomes such as best-corrected visual acuity (BCVA) and spectral-domain optical coherence tomography (SD-OCT) parameters 1 month and beyond following intravitreal anti-VEGF injections in eyes with neovascular AMD [11–13]. However, to our knowledge, no report has been published on the exact measurement of VEGF levels or on clinical outcomes 1 week after intravitreal anti-VEGF injections in neovascular AMD patients. The purpose of this study was to evaluate the concentration of VEGF in the aqueous humor before and after a single intravitreal injection of bevacizumab in eyes with neovascular AMD. Additionally, a correlation among VEGF concentration, BCVA and SD-OCT measurements was investigated.

## Methods

This was a prospective study that measured the aqueous levels of VEGF in eyes with neovascular AMD treated with a single intravitreal injection of bevacizumab at the Retina Department, Public Server Hospital of São Paulo (IAMSPE), São Paulo, Brazil. The present study of the off-label use of bevacizumab and collection of aqueous humor was approved by the Institutional Review Board of Federal University of São Paulo (Reference Number: 215195) and Public Server Hospital of São Paulo (Reference Number: 0115/10). Informed consent was obtained from all study participants.

### Subjects

Aqueous humor samples were obtained from 24 eyes of 24 consecutives patients (12 males and 12 females) with CNV due to neovascular AMD after a single intravitreal injection of bevacizumab.

All study patients had active CNV secondary to AMD that was confirmed by (SD-OCT) and fluorescein angiography (FA). None of participants underwent previous treatment for neovascular AMD. Mean patient age was 74.1 years.

### Ophthalmologic examination

Patients underwent a comprehensive ophthalmologic examination that included best-corrected visual acuity (BCVA), intraocular pressure (IOP) and biomicroscopic examination. Color photographs and fluorescein angiography were performed using a fundus camera (Topcon TRC-50IA; Tokyo Optical Co Ltd, Tokyo, Japan).

Three parameters of OCT (central retinal thickness, macular volume and macular area) were measured using SD-OCT Cirrus 4000, (Carl Zeiss Meditec, Dublin, CA) at the baseline, and at one week, one month and three months follow-up periods. The central thickness (CRT), macular volume (MV) and macular area (MA) were calculated after acquiring a sequence of 128 horizontal sections recorded in the high resolution mode (27,000 A-scans per second). Macular cube 512 × 128 and 5-line raster scans were performed.

### Intravitreal injection and sample collection

Topical anesthesia with tetracaine 1% eye drops was induced prior to injection. Povidone-iodine was applied to the eyelid margins, and a lid speculum was inserted after application of a sterile drape. The intravitreal injection of anti-VEGF, Bevacizumab 1.25 mg/0.05 mL (Avastin, Genentech, South San Francisco, CA), was injected via *pars plana* using a 30-gauge needle into the vitreous cavity in the 24 study eyes with neovascular AMD.

Aqueous humor samples were obtained before the intravitreal injection (baseline), at one week, one month and three months after the treatment (all the patients received a new anti-VEGF injection at this endpoint). Undiluted samples (0.1 mL) were collected via paracentesis using a 30-gauge needle, and then in sterile tubes immediately stored at **−**80 °C until analysis. All of the 96 taps were well tolerated by the patients with no adverse events. Specifically, there was no evidence of lens damage in phakic patients and no hemorrhages, inflammation, or endophthalmitis.

### Measurement of VEGF

The VEGF concentration in the aqueous humor was measured using an enzyme-linked immunometric assay (VEGF (human), Elisa Kit—Ultra-sensitive ELISA, Assay Designs^®^ and Stressgen^®^, Enzo Biochem Inc, Farmingdale, NY). Standard curves for VEGF levels were generated using the reference standard supplied with the kit. The limit of the detectable VEGF-A concentration was 14.04 pg/mL. In the current study, we specifically measured the concentration of VEGF-A in the aqueous humor.

### Statistical analysis

Data were analyzed using commercially available R 3.2.2 software (Redmond, WA). Linear mixed models with a random intercept for subject were used to compare mean VEGF concentration, BCVA, and OCT measurements (CRT, MV, and MA) over time. The time points were before intravitreal bevacizumab injection, at day 7, month 1, and month 3. P < 0.05 was considered statistically significant. The Pearson correlation coefficient was used to correlate VEGF levels, CRT and BCVA at specific time points.

## Results

Intravitreal injection of bevacizumab was administered exclusively to the patients presenting with neovascular AMD. In total, ninety-six taps over time were collected from the 24 study eyes, and there were no complications such as uveitis, lens opacification, nor endophthalmitis.

**Fig. 1:**
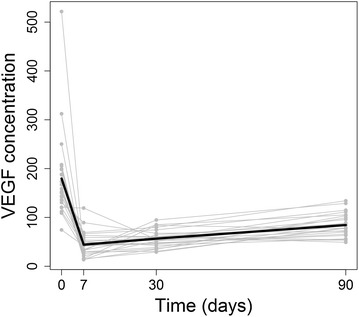
Line graph showing that the mean vascular endothelial growth factor (VEGF) levels were significantly lower at all follow-up periods as compared to the baseline measurement. The lowest VEGF concentration was observed one week after treatment. The *light gray lines* represent the VEGF concentration of each study eye (total of 24 eyes) and the *black line* represents the mean VEGF concentration of all study eyes

At baseline, the mean ± SD concentration of VEGF in the aqueous humor was 179.7 ± 88.4 pg/mL (range: 74.5–521.6 pg/mL). The mean ± SD aqueous concentration of VEGF was 44.2 ± 25.3 pg/mL at 1 week, 56.6 ± 17.8 pg/mL at 1 month and 84.4 ± 23.9 pg/mL at 3 months after intravitreal injection of bevacizumab. At all follow-up periods, the mean VEGF levels were significantly lower as compared to baseline measurement (P < 0.0001, linear mixed models) (Table 1). The lowest VEGF expression when compared to baseline, except for one patient, was observed one week after treatment (Fig. 1). In this patient, the baseline VEGF level persisted during the first week and had a significant decrease at the end of the first month follow-up.

**Table 1:** Vascular endothelial growth facto levels, central macular thickness, and best-corrected visual acuity before and after intravitreal injection of bevacizumab in neovascular age macular degeneration

	Baseline	7d	30d	3 m
VEGF (pg/mL)	179.7 ± 88.369	44.2 ± 25.258	56.6 ± 17.788	84.4 ± 23.378
		*P* < *0.0001*	*P* < *0.0001*	*P* < *0.0001*
BCVA (decimal)	0.044 ± 0.062	0.079 ± 0.079	0.102 ± 0.085	0.084 ± 0.075
		*P 0.0020*	*P* < *0.0001*	*P 0.0005*
SD-OCT				
CRT (μm)	407.0 ± 190.374	307.9 ± 94.128	294.8 ± 104.173	334.3 ± 104.904
		*P 0.0018*	*P 0.0005*	*P 0.0198*
MV (mm^3^)	11.992 ± 3.740	10.700 ± 2.325	10.338 ± 2.286	10.867 ± 2.290
		*P 0.0346*	*P 0.0074*	*P 0.0647*
MA (μm)	332.5 ± 104.318	297.7 ± 64.979	287.4 ± 63.545	299.6 ± 65.462
		*P 0.0420*	*P 0.0091*	*P 0.0543*

*VEGF* vascular endothelial growth factor, *BCVA* best-corrected visual acuity, *SD*-*OCT* spectral-domain optical coherence tomography, *CRT* central retinal thickness, *MV* macular volume, *MA* macular area, *P* P value (comparisons with baseline)

* Linear mixed models were used to compare time points

Mean BCVA ± SD of study patients improved at all follow-up periods (baseline: 0.04 ± 0.06, 1 week: 0.07 ± 0.07, 1 month: 0.10 ± 0.08, 3 months: 0.08 ± 0.07). The improvement of BCVA was greater at 1 month (P < 0.0001, linear mixed models) (Table 1). The mean IOP measurements were not observed to have variation that was statistically different during the study period (baseline: 13.4 ± 0.06, 1 week: 13.5 ± 0.07, 1 month: 12.3 ± 0.08, 3 months: 13.1 ± 0.07).

**Fig. 2:**
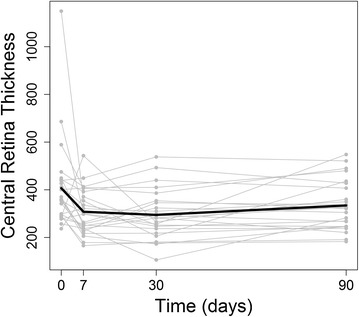
Line graph depicting that the reduction of mean central retinal thickness (CRT) was largest at 1 month in comparison with baseline. The *light gray lines* represent the CRT of each study eye (total of 24 eyes) and the *black line* represents the mean CRT of all study eyes

Regarding SD-OCT measurements, a decrease of CRT (baseline: 407.0 ± 190.3 μm, 1 week: 307.9 ± 94.1 μm, 1 month: 294.8 ± 104.1 μm, 3 months: 334.3 ± 104.9 μm) was also observed following the bevacizumab injection. In comparison with baseline, the reduction of mean CRT was largest at 1 month (baseline mean of 407.0 μm versus 1 month mean of 294.8 μm, P = 0.0005, linear mixed models), while the difference between baseline and 3 months after treatment was smaller (baseline mean of 407.0 μm versus 3 month mean of 334.3 μm, P = 0.02, linear mixed models) (Fig. 2). The SD-OCT MV measurement also had a reduction at all follow-up periods (baseline: 11.9 ± 3.7 mm^3^, 1 week: 10.7 ± 2.3 mm^3^, 1 month: 10.3 ± 2.2 mm^3^, 3 months: 10.8 ± 2.2 mm^3^). The greatest decrease of MV was greatest at 1 month follow-up (P = 0.007, linear mixed models). The greatest SD-OCT MA reduction was also observed at 1 month (baseline mean of 332.5 compared to 1 month mean of 287.4, P = 0.009, linear mixed models) (Table 1).

**Fig. 3:**
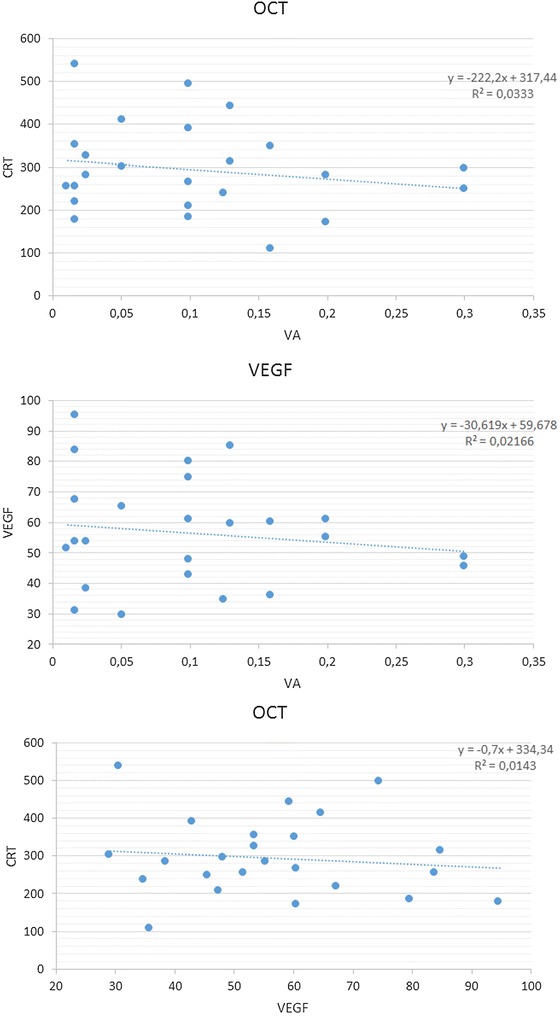
Correlations among VEGF levels, CRT, and best-corrected visual acuity (BCVA) at 1-month follow-up. CRT and VEGF expression were observed to be inversely correlated with BCVA. Conversely, VEGF levels and CRT appeared directly correlated

The results of correlations among VEGF levels, CRT, and BCVA are summarized in Fig. 3. A statistical trend was observed when reduction of CRT and improvement of BCVA, reduction of CRT and decrease of VEGF levels, and improvement of BCVA and decrease of VEGF levels were correlated (r = 0.03, r = 0.01, r = 0.02, respectively).

## Discussion

The development of CNV in patients with neovascular AMD is associated with changes in various angiogenetic factors, and VEGF is one of the most potent stimulators of angiogenesis in the eye [3–5, 8, 24]. Increased VEGF expression in the vascular endothelial cells and retinal pigment epithelial cells has been reported in eyes with active CNV due to neovascular AMD [20], and VEGF suppression by anti-VEGF agents has been shown to be effective for treating CNV secondary to AMD [24]. Although not approved for intravitreal use, off-label bevacizumab therapy rapidly became the most common intravitreal anti-VEGF therapy, and the Comparison of AMD Treatment Trials (CATT) study showed strong evidence that both bevacizumab and ranibizumab have similar efficacy for treating eyes with neovascular AMD, supporting the use of bevacizumab as an effective anti-VEGF therapy [25].

Although the VEGF levels in the vitreous cavity seems to be more predictable than those in the aqueous humor, the collection of vitreous samples before and after intravitreal injections is challenging. It is reported that the VEGF expression in the aqueous humor is considerably related with the VEGF measurement in the vitreous humor. Indeed, the aqueous VEGF has correspondence with neovascular AMD severity [18, 21]. The aim of this study was to measure the aqueous VEGF levels before and after a single intravitreal injection of bevacizumab in eyes with both type 1 and type 2 neovascular AMD. Clinical correlation among VEGF levels, BCVA and SD-OCT parameters was also investigated.

Our data showed that the mean VEGF levels were statistically significant lower compared to the baseline measurement at all follow-up evaluations. The lowest aqueous VEGF expression (44.2 pg/mL) was observed 1 week after the intravitreal bevacizumab injection. The mean VEGF expression increased to 56.6 pg/mL at 1 month and to 84.4 pg/mL at 3 months, which was almost twice the level at the 1-week evaluation. In addition, mean BCVA of the study patients improved at all follow-up periods with the greatest improvement (0.10 ± 0.08) observed 1 month after intravitreal bevacizumab injection. Reduction in the measurements of the study SD-OCT parameters (CRT, MV and MA) was observed over time following the intravitreal bevacizumab injection, and the greatest CRT, MV and MA reduction occurred at 1 month follow-up period. The correlations performed between BCVA and VEGF expression and between BCVA and CRT showed a statistical trend to improved BCVA in the setting of lower VEGF levels and more reduced CRT. The VEGF level at 3-month after the injection was well suppressed compared with that at the baseline while CRT increased in many cases. This may be due to the expression of other inflammatory biomarkers following the decrease of VEGF concentration that may result in retinal leakage and increase of central retinal thickness. Hence, an increased CRT may be observed in the setting of lower VEGF concentration.

Clinical prospective studies have reported the effect of anti-VEGF therapies on the aqueous and vitreous VEGF levels [23, 26–29]. In such studies, early reduction of VEGF levels was demonstrated in proliferative diabetic retinopathy, retinopathy of prematurity and neovascular AMD after intravitreal bevacizumab injection. Sawada et al. [23] measured the aqueous VEGF levels in patients with proliferative diabetic retinopathy after intravitreal bevacizumab injection and observed a significant decrease in the level of aqueous VEGF at the 1-week follow-up compared to baseline. However, the precise VEGF concentration 1 week after intravitreal bevacizumab injection was not determined because the VEGF concentration decreased to less than 31 pg/mL (the limit of detection) and the limit of measurement of the enzyme-linked immunosorbent assay used in this study was 31 pg/mL. Matsuyama et al. [28] also observed that 1.25 mg of intravitreal bevacizumab significantly decreased aqueous VEGF levels by 7 days in patients with proliferative diabetic retinopathy and neovascular glaucoma. Dell’Omo et al. [29] reported that, regardless of the type of AMD neovascularization (type 1, 2 or 3 neovascularization), aqueous VEGF levels decreased significantly 1 month after the intravitreal bevacizumab injection in comparison with both the baseline measurements and the values recorded in age-matched controls. This decrease in the VEGF level was maintained at 2 months after administration of a second intravitreal bevacizumab injection 30 days after the initial injection. Additionally, a reduction of central macular thickness was also observed in all study groups in comparison with baseline. Nonobe et al. [30] measured the aqueous VEGF levels shortly (4 days) after intravitreal bevacizumab injection in eyes with advanced retinopathy of prematurity and found a marked reduction of VEGF concentration. All of these cited studies corroborate our findings of decreased VEGF levels at 1 week and 1 month. In contrast to these previous reports, we administered only one intravitreal bevacizumab injection and precisely measured the VEGF expression 1 week, 1 and 3 months after the injection in patients with type 1 and type 2 neovascular AMD.

In the current study, the lowest level of VEGF was observed 1 week after the intravitreal bevacizumab injection, and the greatest BCVA improvement and SD-OCT parameters (CRT, MV and MA) reduction occurred 1 month after the injection. The intravitreal half-live of low doses of bevacizumab (1.25 and 1.5 mg) is reported to be approximately 7 days [31–34] which may explain the greatest reduction of aqueous VEGF expression at the 1-week evaluation. Also, the minimum bevacizumab concentration (500 ng/ml) to block VEGF-A effects is observed up to 48 days and the concentration above the half maximal inhibitory concentration (22 ng/ml) could be maintained for approximately 78 days after 1.25 mg intravitreal injection in eyes with choroidal neovascularization [34]. It corroborates our finding that at 1 month and 3 months follow up periods the VEGF-A levels remained significantly reduced. Therefore, our results may agree with the proposed dosing of bevacizumab every 2 weeks that resulted in increased trough binding levels compared with monthly dosing. The shorter interval between anti-VEGF injections, such as biweekly injections, may benefit patients who respond poorly to monthly therapy [31]. Our data are also in accordance with other studies that have observed the greatest BCVA improvement and SD-OCT parameters reduction 1 month after the injection [11–13].

## Conclusions

The present study had some limitations, i.e., its small sample size and the short follow-up period. Further, the neovascular disease activity was not correlated with the aqueous VEGF measurements. In conclusion, we reported that the greatest reductions in the aqueous VEGF expression occurred 1 week after the intravitreal bevacizumab injection, and the greatest BCVA improvement and SD-OCT parameters reduction occurred 1 month after the injection. Therefore, the aqueous VEGF levels might be a potential marker for the neovascular activity in eyes with AMD.

## Abbreviations

AMD: age-related macular degeneration
BCVA: best-corrected visual acuity
CNV: choroidal neovascularization
CRT: central retinal thickness
FA: fluorescein angiography
IOP: intraocular pressure
MA: macular area
MV: macular volume
SD-OCT: spectral-domain optical coherence tomography
VEGF: vascular endothelial growth factor
